# Supplementary material for: Rietveld Quantitative Phase Analysis of Oil Well Cement: In Situ Hydration Study at 150 Bars and 150 °C
Source: Materials (Basel). 2019 Jun 12;12(12):1897. doi: 10.3390/ma12121897 (PMC6631950; doi:10.3390/ma12121897)
Supplement: Supplementary file 1 [file materials-12-01897-s001.pdf]

Rietveld quantitative phase analysis of Oil Well Cement: *in situ* hydration study at 150 bars and 150ºC

Edmundo Fraga,[1,3] Ana Cuesta,[2] Jesus D. Zea-Garcia,[2] Angeles G. De la Torre,[2] Armando Yáñez-Casal,[3] Miguel A. G. Aranda[1,2,]*

[1]ALBA Synchrotron, Carrer de la Lum, 2-26, 08290 Cerdanyola del Vallès, Barcelona, Spain. [2]Departamento de Química Inorgánica, Cristalografía y Mineralogía. Universidad de Málaga, 29071 Málaga, Spain. [3] Universidade da Coruña, Dpto. Ingeniería Industrial II, E-15403 Ferrol, A Coruña, Spain
*Correspondence e-mail: g_aranda@uma.es

This supporting information includes:

Extended materials and methods

Table S1. Elemental composition of the Oil Well Portland cement Class G HSR used in this study.

Table S2. Direct Rietveld Quantitative Phases Analysis of Oil Well paste after selected hydration times under 150 bars and 150 ºC.

Figure S1. Particle size distribution and cumulative volumetric particle size distribution for the pristine oil well cement.

Figure S2. Calorimetric data. (a) Heat flow calorimetry curves and (b) Cumulative heat released for OWC paste (w/c mass ratio of 0.47) –blue–, OWC paste (w/c mass ratio of 0.35) –green– and a normal white Portland cement (w/c mass ratio of 0.35) used as reference –red–.

Figure S3. 2D synchrotron powder diffraction patterns of the oil well cement (a) anhydrous, and paste hydrated at 150 bars and 150ºC for (b) 1 hour and 38 minutes and (c) 11 hours and 22 minutes. Some diffractions spots arising from the sapphire tube are highlighted.

Extended materials and methods

BET (Brunauer–Emmett–Teller): The specific surface area of the oil well cement was measured by nitrogen sorption applying the BET methodology. The measurement was carried out in an automatic MICROMERITICS ASAP 2020 (Micromeritics Instrument Corp, GA, USA). Isotherms at low partial pressures of the inert gas (N_2, at room temperature) were used to determine specific surface areas. The total surface area of the powder was calculated using the Langmuir theory and the BET generalization.

Table S1. Elemental composition of the Oil Well Portland cement Class G HSR used in this study.

XRF*[#]** ***Oxide, wt%	***OWC***
CaO	64.4
SiO_2	21.2
Al_2O_3	4.3
Fe_2O_3	5.5
SO_3	2.7
K_2O	0.7
Na_2O	0.1
MgO	0.9
P_2O_5	0.1

[#] Cl content: 0.007 wt%

Table S2. Direct Rietveld Quantitative Phases Analysis results for the Oil Well Cement paste after selected hydration times under 150 bars and 150 ºC.

Phases /wt%	t_0	9'	26'	53'	1h38'	3h15'	5h20'	7h19'	9h13'	11h22'	13h54'
C_3S [1]	61.2(1)	59.8(1)	57.5(1)	51.8(1)	33.3(2)	19.1(2)	12.2(3)	8.4(2)	6.5(3)	5.5(3)	4.1(3)
β-C_2S [2]	14.6(2)	14.8(1)	16.5(2)	17.2(2)	21.2(3)	23.2(3)	22.7(3)	21.9(3)	20.4(3)	18.4(3)	15.7(3)
C_4AF [3]	16.6(1)	16.6(1)	15.0(1)	15.8(1)	17.2(2)	19.0(2)	20.2(2)	20.8(2)	21.2(2)	21.8(2)	22.0(2)
o-C_3A [4]	3.4(1)	3.3(1)	1.9(1)	-	-	-	-	-	-	-	-
$C\bar{S}H_2$ [5]	4.2(2)	4.6(1)	-	-	-	-	-	-	-	-	-
$C\bar{S}H_{0.5}$ [6]	-	-	1.8(1)	-	-	-	-	-	-	-	-
CH [7]	-	-	0.5(1)	5.2(1)	15.4(1)	23.7(1)	28.8(1)	31.6(1)	33.4(1)	34.4(1)	35(1)
AFt [8]	-	0.9(1)	1.0(1)	-	-	-	-	-	-	-	-
Jaffeite [9]	-	-	-	-	-	-	-	-	-	-	0.8(2)
Katoite [10]	-	-	5.8(1)	10.0(2)	12.9(2)	15.0(2)	16.1(2)	16.8(2)	17.0(2)	17.0(2)	16.8(2)
α-C_2SH [11]	-	-	-	-	-	-	-	0.5(3)	1.5(3)	2.9(3)	5.6(3)
R_{WP} /%	2.0	1.3	1.4	2.1	2.1	2.1	2.0	1.9	1.8	1.8	1.8
$R_F(C_3S)$ /%	5.5	3.7	3.6	5.5	7.2	9.4	11.3	12.7	13.7	14.6	14.8

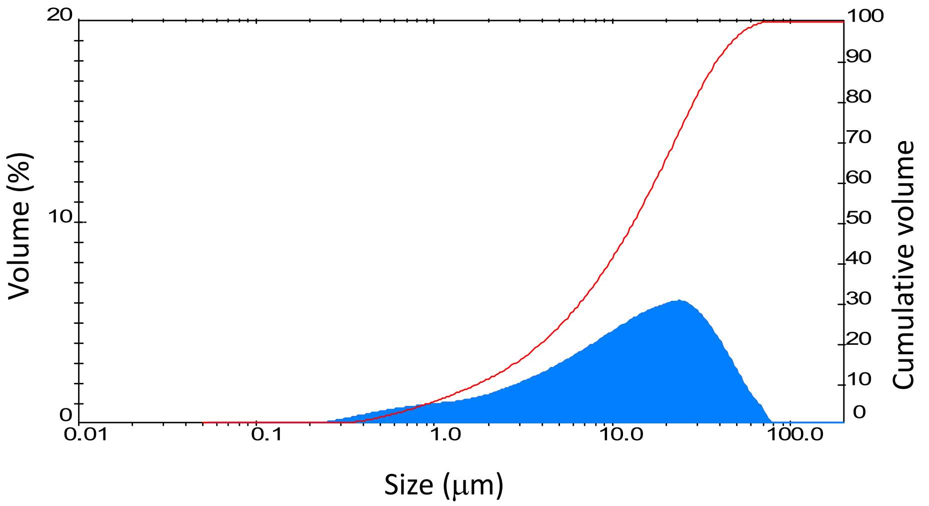

Figure S1. Particle size distribution and cumulative volumetric particle size distribution for the pristine oil well cement.

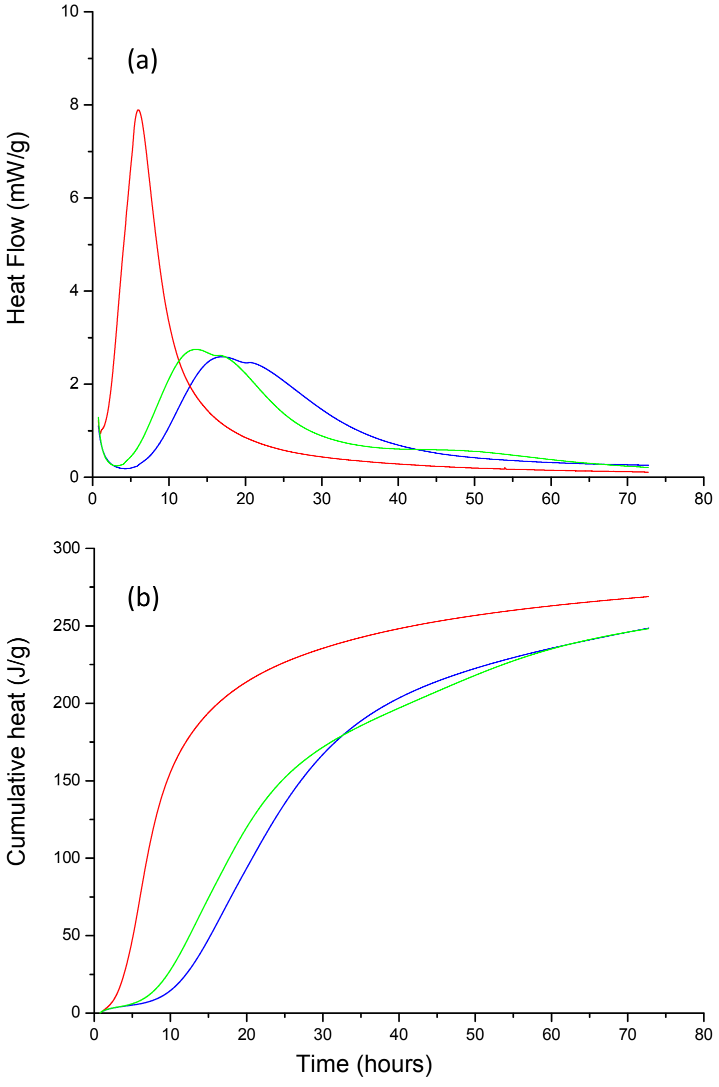

Figure S2. Calorimetric data. (a) Heat flow calorimetry curves and (b) Cumulative heat released for OWC paste (w/c mass ratio of 0.47) –blue–, OWC paste (w/c mass ratio of 0.35) –green– and a normal white Portland cement (w/c mass ratio of 0.35) used as reference –red–.

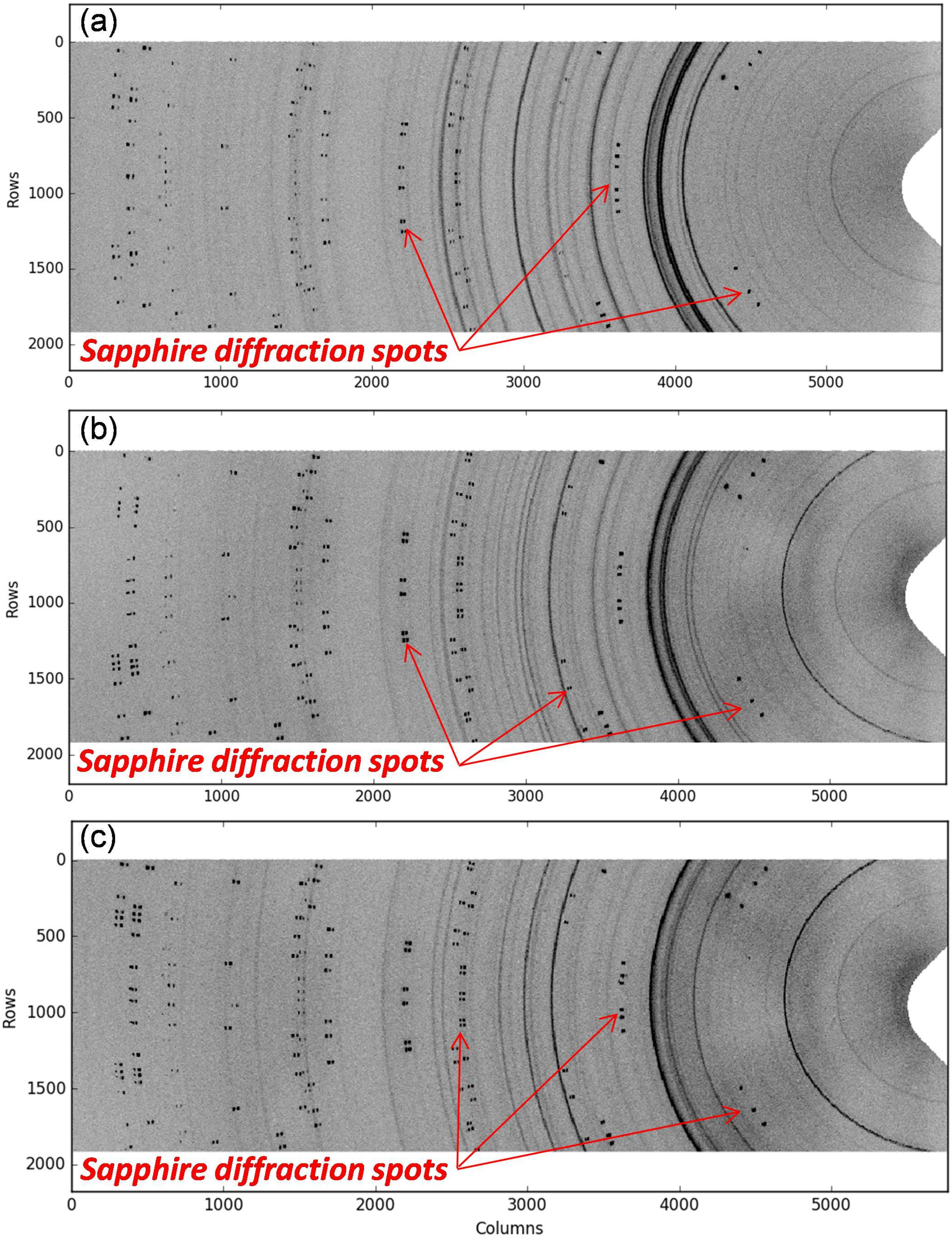

Figure S3. 2D synchrotron powder diffraction patterns of the oil well cement (a) anhydrous, and paste hydrated at 150 bars and 150ºC for (b) 1 hour and 38 minutes and (c) 11 hours and 22 minutes. Some diffractions spots arising from the sapphire tube are highlighted.

References

1. De La Torre, A.G.; Bruque, S.; Campo, J.; Aranda, M.A.G. The superstructure of C3S from synchrotron and neutron powder diffraction and its role in quantitative phase analyses. *Cem. Concr. Res.* **2002**, *32*, 1347–1356.
2. Mumme, W.G.; Hill, R.J.; Bushnell-Wye, G.; Segnit, E.R. Rietveld crystal structure refinements, crystal chemistry and calculated powder diffraction data for the polymorphs of dicalcium silicate and related phases. *Neues Jahrb. fuer Mineral.* **1995**, *169*, 35–68.
3. Colville, A.A.; Geller, S. The Crystal Structure of Brownmillerite, Ca2FeAlO5. *Acta Cryst* **1971**, *27*, 5.
4. Takéuchi, Y.; Nishi, F. Crystal-chemical characterization of the 3CaO·Al2O3—Na2O solid-solution series. *Zeitschrift für Krist. - Cryst. Mater.* **1980**, *152*, 259–308.
5. De la Torre, A.G.; López-Olmo, M.-G.; Álvarez-Rua, C.; García-Granda, S.; Aranda, M.A.G. Structure and microstructure of gypsum and its relevance to Rietveld quantitative phase analyses. *Powder Diffr.* 2004, *19*, 240–246.
6. Bezou, C.; Nonat, A.; Mutin, J.-C.; Christensen, A.N.; Lehmann, M.S. Investigation of the Crystal Structure of γ-CaSO4, CaSO4 · 0.5 H2O, and CaSO4 · 0.6 H2O by Powder Diffraction Methods. *J. Solid State Chem.* **1995**, *117*, 165–176.
7. Petch, H.E.; IUCr The hydrogen positions in portlandite, Ca(OH)2, as indicated by the electron distribution. *Acta Crystallogr.* **1961**, *14*, 950–957.
8. Goetz-Neunhoeffer, F.; Neubauer, J. Refined ettringite (Ca6Al2(SO4)3(OH)12·26H2O) structure for quantitative X-ray diffraction analysis. *Powder Diffr.* **2006**, *21*, 4–11.
9. Yamnova, N.A.; Sarp, K.; Egorov-Tismenko, Y.K.; Pushcharovskii, D.Y. Crystal structure of jaffeite. *Crystallogr. Reports* **1993**, *38*, 464–467.
10. Sacerdoti, M.; Passaglia, E. The crystal structure of katoite and implications within the hydrogrossular group of minerals. *Bull. Minéralogie* **1985**, *108*, 1–8.
11. Yano, T.; Urabe, K.; Ikawa, H.; Teraushi, T.; Ishizawa, N.; Udagawa, S.; IUCr Structure of α-dicalcium silicate hydrate. *Acta Crystallogr. Sect. C Cryst. Struct. Commun.* **1993**, *49*, 1555–1559.
